# PD-1 Impairs CD8^+^ T Cell Granzyme B Production in Aged Mice during Acute Viral Respiratory Infection

**DOI:** 10.4049/immunohorizons.2300094

**Published:** 2023-11-28

**Authors:** Olivia B. Parks, Danielle Antos, Taylor Eddens, Sara Walters, Monika Johnson, Tim D. Oury, Rachel A. Gottschalk, John J. Erickson, John V. Williams

**Affiliations:** * Division of Infectious Diseases, Department of Pediatrics, University of Pittsburgh School of Medicine, Pittsburgh, PA; † Division of Pulmonology, Department of Pediatrics, University of Pittsburgh School of Medicine, Pittsburgh, PA; ‡ Division of Allergy/Immunology, Department of Pediatrics, University of Pittsburgh School of Medicine, Pittsburgh, PA; § Department of Pathology, University of Pittsburgh School of Medicine, Pittsburgh, PA; ¶ Department of Immunology, University of Pittsburgh School of Medicine, Pittsburgh, PA; ‖ Division of Neonatology and Pulmonary Biology, Department of Pediatrics, Cincinnati Children’s Hospital Medical Center, University of Cincinnati School of Medicine, Cincinnati, OH; # Institute for Infection, Inflammation, and Immunity in Children (i4Kids), Pittsburgh, PA

## Abstract

CD8^+^ T cell dysfunction contributes to severe respiratory viral infection outcomes in older adults. CD8^+^ T cells are the primary cell type responsible for viral clearance. With increasing age, CD8^+^ T cell function declines in conjunction with an accumulation of cytotoxic tissue-resident memory (T_RM_) CD8^+^ T cells. We sought to elucidate the role of PD-1 signaling on aged CD8^+^ T cell function and accumulation of CD8^+^ T_RM_ cells during acute viral respiratory tract infection, given the importance of PD-1 regulating CD8^+^ T cells during acute and chronic infections. PD-1 blockade or genetic ablation in aged mice yielded improved CD8^+^ T cell granzyme B production comparable to that in young mice during human metapneumovirus and influenza viral infections. Syngeneic transplant and adoptive transfer strategies revealed that improved granzyme B production in aged *Pdcd1*^−/−^ CD8^+^ T cells was primarily cell intrinsic because aged wild-type CD8^+^ T cells did not have increased granzyme B production when transplanted into a young host. PD-1 signaling promoted accumulation of cytotoxic CD8^+^ T_RM_ cells in aged mice. PD-1 blockade of aged mice during rechallenge infection resulted in improved clinical outcomes that paralleled reduced accumulation of CD8^+^ T_RM_ cells. These findings suggest that PD-1 signaling impaired CD8^+^ T cell granzyme B production and contributed to CD8^+^ T_RM_ cell accumulation in the aged lung. These findings have implications for future research investigating PD-1 checkpoint inhibitors as a potential therapeutic option for elderly patients with severe respiratory viral infections.

## Introduction

Lower respiratory infections contribute to increased morbidity and mortality in adults age >65 y (1). Human metapneumovirus (HMPV) is a leading cause of acute lower respiratory infection and occurs with a similar incidence among older adults as influenza and respiratory syncytial virus (2–5). Despite universal exposure to HMPV and 100% seropositivity worldwide by age 5 y, recurrent infections occur throughout life, especially in adults aged >65 y, in whom reinfection can cause severe respiratory disease (2–5).

Increased age is associated with a widespread decline in immune cell function and a subsequent increase in basal proinflammatory cytokine production, referred to as “inflammaging” (6, 7). In addition, aged mice and humans have increased levels of proinflammatory GZMK^+^ CD8^+^ T cells (8), terminally differentiated CD8^+^ T cells expressing thymocyte selection–associated high-mobility group box protein (TOX) and eomesodermin (EOMES) (8–10), as well as tissue-resident memory (T_RM_) CD8^+^ T cells that propagate lung inflammation and fibrosis (11).

CD8^+^ T cells are the primary mediators of HMPV clearance (12–15). HMPV infection causes virus-specific CD8^+^ T cells to upregulate inhibitory receptors such as programmed cell death-1 (PD-1) and lymphocyte activation gene 3 (LAG-3) (12). PD-1 expression impairs CD8^+^ T cell degranulation and cytokine production during HMPV infection (12–15). Studies in aged mice revealed that virus-specific CD8^+^ T cells and antiviral functions, particularly granzyme B production, decline with age, impacting the immune response to respiratory viruses (16–19). We found that aged mice infected with HMPV displayed severe clinical disease, delayed viral clearance, and exacerbated lung inflammation compared with young mice (20). Aged HMPV-infected mice also generated fewer virus-specific CD8^+^ T cells that possessed a terminally differentiated phenotype characterized by a loss of expression of transcription factor 7 (TCF7) and increased expression of TOX and EOMES—TCF^−^ TOX^+^ EOMES^+^—and displayed significantly decreased granzyme B production (8–10, 20). Upon HMPV rechallenge, aged mice exhibited severe disease and accumulated cytotoxic CD8^+^ T_RM_ cells (21).

PD-1 blockade has successfully been used to rejuvenate exhausted CD8^+^ T cells in lymphocytic choriomeningitis virus chronic infection (22, 23) and cancer models (24, 25). Immune checkpoint inhibitors targeting PD-1 signaling have comparable efficacy in both young adults and elderly humans with metastatic solid tumors, indicating that the aged immune system has the capacity to respond to PD-1 blockade (26). In young mice infected with HMPV, PD-1 neutralization or genetic ablation resulted in improved CD8^+^ T cell function and improved viral clearance (12–15). In addition, abrogating PD-1 signaling during influenza virus infection improved viral clearance and increased CD8^+^ T cell production of IFN-γ and granzyme B (27). PD-1 signaling has been implicated in optimal CD8^+^ memory T cell formation following influenza infection, suggesting that the timing of PD-1 blockade during acute viral infection is important to enhance CD8^+^ T cell function while not impairing memory formation (28). However, the effects of PD-1 blockade on the aged CD8^+^ T cell response and T_RM_ cell formation during HMPV infection are poorly understood.

In the present study, we sought to elucidate the role of PD-1 signaling on virus-specific CD8^+^ T cell function and memory T_RM_ cell formation in aged mice. We found that aged *Pdcd1*^−/−^ mice had improved CD8^+^ T cell granzyme B production during either HMPV or influenza infection. This increase in function was primarily cell intrinsic because aged *Pdcd1*^−/−^ CD8^+^ T cells transplanted into young congenically marked recipients had improved antiviral function compared with aged wild-type (WT) CD8^+^ T cells. In addition, aged *Pdcd1*^−/−^ mice did not accumulate cytotoxic CD8^+^ T_RM_ cells at day 40 postinfection (p.i.), which was in contrast to the detrimental accumulation of cytotoxic CD8^+^ T_RM_ cells in aged WT mice. Aged mice treated with PD-1 blockade and rechallenged with HMPV 14 mo after primary infection lost less weight and had a trend toward improved CD8^+^ T cell IFN-γ production as compared with isotype control mice, but they did not accumulate as many CD8^+^ CD69^+^ CD103^+^ memory T cells. Taken together, these results suggest that therapeutic PD-1 blockade in the aged host may improve CD8^+^ T cell function during respiratory viral infections.

## Materials and Methods

### Mice and viral infection

C57BL/6 (B6), congenic CD45.1, and *Rag*^−/−^ mice were purchased from The Jackson Laboratory. *Pdcd1*^−/−^ mice were obtained with permission from Dr. Tasuku Honjo (Kyoto University, Kyoto, Japan). All animals were bred and maintained in specific pathogen-free conditions in accordance with the University of Pittsburgh Institutional Animal Care and Use Committee. Six- to 7-wk-old and 10-mo-old *Pdcd1*^−/−^ and age-matched B6 mice were used in all experiments involving *Pdcd1*^−/−^ mice. In select rechallenge experiments, 70–71-wk-old B6 animals were used. HMPV (strain TN/94-49, genotype A2) was grown and titered in LLC-MK2 cells as described (29). Influenza virus strain A/34/PR/8 (PR8) was grown in MDCK cells and titered on LLC-MK2 cells (12). For all experiments, mice were anesthetized with isoflurane in a heated chamber and infected via the orotracheal route with 2.0 × 10^6^ PFU HMPV, 500 PFU PR8, or sterile PBS in a 100-μl volume. Mock-infected mice were infected under the same conditions with sterile PBS. Viral titers were measured by plaque assay as described (29, 30).

### Ab treatment

On the two days prior to infection and days 1, 2, and 5 p.i., aged and young B6 mice were injected i.p. with 200 μg in sterile PBS of αPD-1 (Bio X Cell, catalog no. BE0033-2) or rat IgG2a isotype control (Bio X Cell, catalog no. BE0089) via i.p. injections.

### Bone marrow transplant

#### Irradiation

One day prior to bone marrow (BM) transplant, an X-ray source (MultiRad 350, Precision X-Ray Irradiation) was used to condition recipient mice with 10–11 cGy total body irradiation in two split doses 4 h apart. Irradiated mice were placed on an immunocompromised rack in the animal facility and given sterile food and water.

#### Bone marrow single-cell suspension

Femurs and tibias from *Rag1*^−/−^ mice were harvested, tissue was removed, and clean cuts were made at either bone end. Using a 25-gauge needle, marrow was flushed from the bone into a conical tube using DMEM with 10% FBS, 1% penicillin-streptomycin antibiotics, 1% l-glutamine, 1% MEM nonessential amino acids, and 0.1% 50 mM 2-ME (D-10 medium). BM was spun down at 350 × *g* for 5 min, and the cell pellet was filtered through a 70-μm strainer, washed twice with 5 ml sterile PBS, and counted on a BD Accuri cytometer.

#### B and T cell magnetic column selection

For B and T lymphocyte magnetic column selection, spleens and lymph nodes (inguinal, peritoneal, and submandibular) were collected from B6 and congenic CD45.1 donor mice aged either 6–7 wk or 70–71 wk. All lymph nodes and each spleen per mouse were passed through a 70-µm strainer, spun down at 350 × *g* for 5 min, pooled together through a 40-µm strainer, and spun down again at 350 × *g* for 5 min. The spleen/lymph node single-cell suspensions from two mice were combined in 900 μl autoMACS buffer, incubated with CD90.2 microbeads or CD19-biotin and biotin-labeled microbeads, and separated via a liquid separation column (Miltenyi Biotec, 130-042-401) per Miltenyi Biotec instructions. Plunge was collected from the columns, then spun down at 350 × *g* for 5 min, and the cell pellet was washed twice with 5 ml sterile 1× PBS. Cells were counted on a BD Accuri cytometer.

#### Tail vein injections

A total of 1 × 10^7^ T, B, and *Rag1*^−/−^ BM cells were resuspended per 1 ml of sterile PBS, followed by injection of 200 μl cells (2 × 10^6^ of each cell type) into irradiated recipient mice via tail vein injection.

### CD4^+^ and CD8^+^ T cell adoptive transfer

CD4 and CD8 biotinylated Abs along with anti-biotin microbeads were used for positive selection of CD4 and CD8 subsets of T cells for use in combination experiments. Totals of 1 × 10^6^ CD4 and 1 × 10^6^ CD8 T cells were combined per mouse, with the remainder of the cell populations as outlined above.

### Mixed bone marrow chimera

The BM single-cell suspension was harvested as outlined above from aged CD45.2 and young CD45.1 donors. BM was injected via tail vein injection into lethally irradiated aged CD45.2 and young CD45.1 recipients in a 1:1 ratio.

### CD45.2 IV labeling

Mice were administered 4 μg CD45.2-BUV496 (BD, catalog no. 741092) in 200 μl sterile PBS via tail vein injection and were euthanized 3 min after injection as previously described (11).

### IFN-γ ELISPOT assay

The ELISPOT assay was performed as previously described (12, 31). In select experiments, 10 μg αPD-1 (Bio X Cell, catalog no. BE0033-2) or rat IgG2a isotype control (Bio X Cell, catalog no. BE0089) was added to ELISPOT wells along with HMPV N_11-18_ peptide. Influenza NP366 peptide (GenScript Peptide Sequence: ASNENMETM) served as a control.

### IgG HMPV ELISA

One microgram of TN/94-49 HMPV viral stock per well was diluted in 1× ELISA coating buffer (BioLegend, catalog no. 421701) and plated overnight at 4°C. The remainder of the ELISA was performed as in (32).

### Flow cytometry staining

Flow cytometry staining was performed as described previously (20, 21). In brief, mice were euthanized, and their right lungs were harvested. The lung was cut into 2-mm segments using scissors, resuspended in RPMI/10% FBS, and incubated for 1 h at 37°C with DNase and collagenase. After digestion, the lung was filtered through 70-μm filters, then spun at 1500 rpm for 5 min, and the pellet was resuspended in 2 ml ACK lysis buffer (Life Technologies, A10492-01) for 1 min. Ten milliliters of RPMI/10% FBS was added after ACK lysis, and cells were spun at 1500 rpm for 5 min. Cells then underwent either tetramer staining or ex vivo peptide stimulation.

#### Tetramer staining

Cells were incubated with 1:2000 dasatinib in 1× PBS/1% FBS (FACS) for 30 min before adding allophycocyanin-conjugated N_11-18_ 1:200 in FACS/dasatinib for 90 min. Cells were then spun down at 1500 rpm for 3 min and washed once with FACS buffer.

#### Ex vivo peptide stimulation

Cells (100 μl) were added to a flat-bottomed 96-well tissue culture plate. The following were added to cells: 100 μl 200 μM N11 HMPV peptide or NP366 for irrelevant control diluted 1:10 in RPMI/10% FBS, 6μl CD107a-PE, and 22 μl brefeldin A (BD, catalog no. 51-2301KZ)/monensin (BD, catalog no. 2092KZ). In addition, 1:1000 PMA/ionomycin instead of peptide was added to one aliquot of cells as a positive control. Cells were incubated for 5 h at 37°C.

#### For both conditions

After either tetramer staining or peptide stimulation, cells were stained with Live/Dead dye 1:1000 in PBS for 12 min, washed once with PBS, and blocked with αCD16/32 Fc block (Tonbo Biosciences, catalog no. 70-0161-M001) 1:100 in FACS buffer for 10 min. For surface staining, cells were stained with surface Ab 1:100 in BD Brilliant Stain Buffer (BD, catalog no. 566349) buffer for 30 min at 4°C. Cells were spun at 1500 rpm for 3 min and washed once with FACS buffer.

#### Intracellular cytokine staining

Following staining for surface markers, cells were fixed for 30 min with the eBioscience Foxp3/Transcription Factor Staining Buffer Set (Thermo Fisher, 00-5523-00) at 4°C, spun at 1640 rpm for 3 min, washed once with Foxp3 Fix/Perm Buffer, and stained with 6 µl/Ab in Foxp3 Fix/Perm Buffer for 1 h at 4°C. Cells were spun at 1640 rpm for 3 min, washed once with FACS buffer, resuspended in FACS buffer, and stored in the dark at 4°C until analyzed on the Cytek Aurora multispectral flow cytometer.

#### Intracellular transcription factor staining

For transcription factor staining, cells were fixed for 18 h in Foxp3 Fix/Perm buffer at 4°C. After fixation/permeabilization, cells were washed once with Foxp3 Fix/Perm Buffer and stained with 2.5 μl Ab in Foxp3 Fix/Perm Buffer for 1 h at 4°C.

After intracellular staining, cells were spun down at 1640 rpm for 3 min, washed once with FACS buffer, resuspended in FACS buffer with 100 μl BioLegend Precision Count Beads (BioLegend, catalog no. 424902), and run on the Cytek Aurora multispectral flow cytometer. Fluorescence minus one controls were used for all inhibitory receptors and transcription factors. For HMPV tetramer staining, influenza NP366-APC tetramers were used as irrelevant controls. Any irrelevant tetramer background staining was subtracted from the final tetramer frequency. Unstained cells from each experiment were fixed for 20 min in 2% PFA and used on the flow cytometer to minimize autofluorescence. Data analysis was performed with FlowJo (version 10.8.1). A full list of Abs used in all experiments is shown in Supplemental Fig. 4G.

### Single-cell RNA-sequencing data analysis

The CD8^+^ T cell single-cell RNA-sequencing (scRNAseq) dataset from lungs of mice and humans was kindly shared with us by Maxim Artyomov (https://www.synapse.org/#!Synapse:syn22255433/wiki/604556) and was generated as described in (8).

### Quantitative RT-PCR

Quantitative RT-PCR was performed as in (12). All values were normalized to the housekeeping gene *Hprt*. Experimental WT and *Pdcd1*^−/−^ lung homogenate samples were reported as relative expression to the housekeeping gene (*Hprt*). Samples with cycle threshold values less than 40 were considered positive.

### Histopathologic score

Histopathologic scoring was performed as in (20, 21). Ten percent formalin was injected into a section of the lower left lung lobe and stored in 10% formalin in histology cassettes (Fisher Scientific, B851000WH). Tissue sections were stained with H&E by the University of Pittsburgh Medical Center Children’s Hospital of Pittsburgh Histology Core, and slides were imaged and scored at 200× magnification. Scoring criteria per field were as follows: 0, no inflammation; 1, 75% inflammation. To generate the histopathologic score, the score for each sample was added and divided by the total number of fields analyzed.

### Statistical analysis

Data analysis was performed using Prism version 9.0 (GraphPad Software). Comparisons between two groups were performed using an unpaired two-tailed Student *t* test or Mann–Whitney test as appropriate. Multiple-group comparisons were performed using one-way or two-way ANOVA as appropriate with correction for multiple comparisons (Dunnett test). A *p* value less than 0.05 was considered significant. Error bars in each graph represent SEM.

### Study approval

All animals were maintained in accordance with the *Guide for the Care and Use of Laboratory Animals* (NIH publication no. 85-23, revised 1985) and were handled according to protocols approved by the University of Pittsburgh Institutional Animal Care and Use Committee.

## Results

### Aged CD8^+^ T cells upregulate PD-1 at baseline and during HMPV infection

**FIGURE 1.**
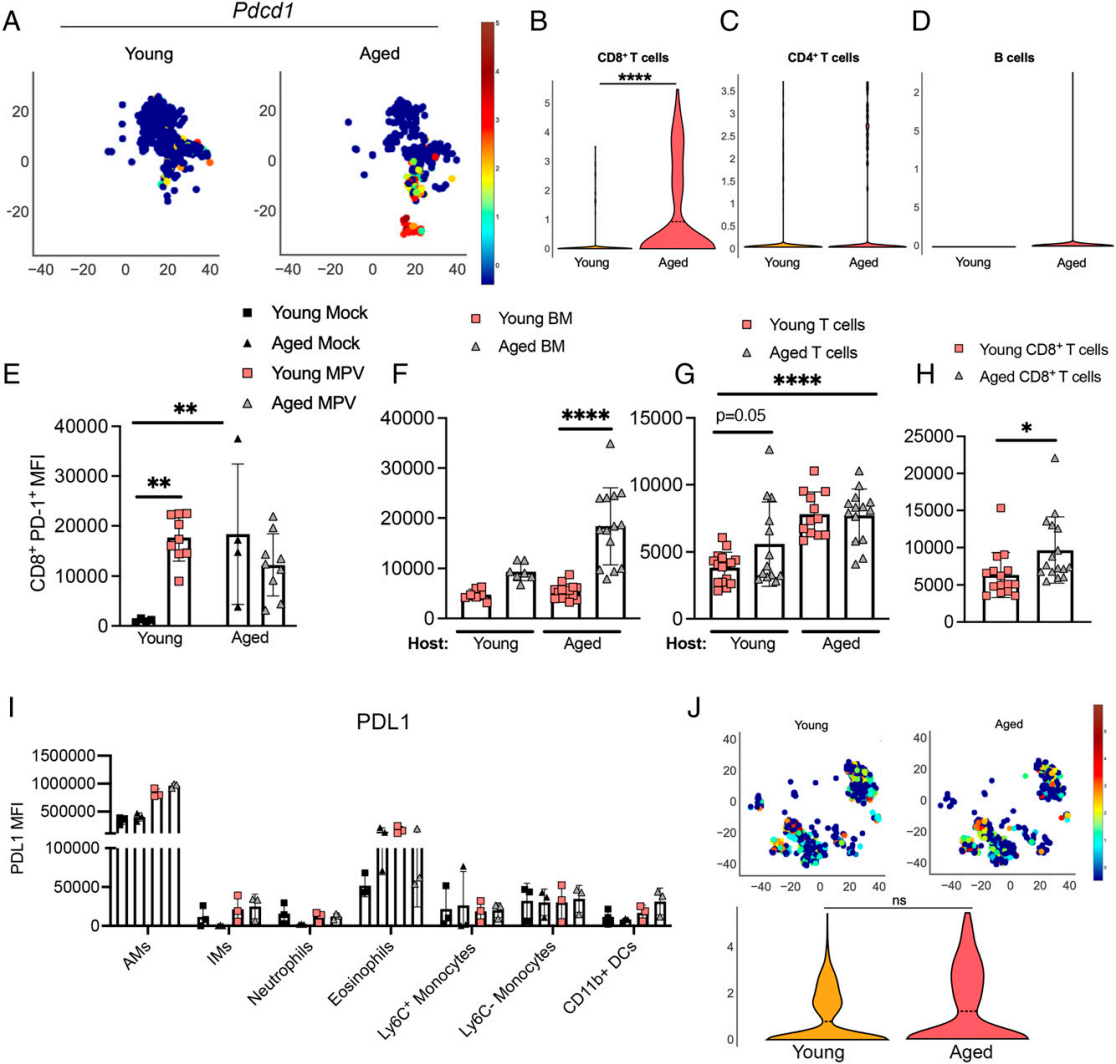
Aged CD8^+^ T cells upregulate PD-1 at baseline and during HMPV infection. (**A**) Heat map of *Pdcd1* on lung CD8^+^ T cells from uninfected, naive aged and young mice. (**B**–**D**) Violin plot of *Pdcd1*^−/−^ expression from uninfected, naive aged and young mice on lung CD8^+^ T, CD4^+^ T, and B cells, respectively. (**E**) Mean fluorescence intensity (MFI) of PD-1 expression on CD8^+^ T cells in young and aged mock-infected or infected mice. (**F**–**H**) MFI of PD-1 expression on lung CD8^+^ T cells in day 7 p.i. in (F) aged/young mixed BM chimera model, (G) syngeneic transplant of bulk aged or young T cells along with young B cells and *Rag1*^−/−^ BM, and (H) adoptive transfer of aged or young CD8^+^ T cells into young *Rag1*^−/−^ recipients. (**I**) PDL1 MFI expression on lung innate immune cells at day 1 p.i. (**J**) Heat map of PD-L1 (i.e., *CD274*) expression in lung myeloid cell cluster in uninfected, naive young and aged mice (top) and violin plot (bottom). Each data point represents one individual mouse. Data in (E) represent three experimental replicates, one to three mouse/group. Data in (F) represent two experimental replicates, three to seven mice/group. Data in (G) represent four experimental replicates, three to five mice/group. Data in (H) represent two experimental replicates, six or seven mice/group. Data in (I) represent one experimental replicate, three mice/group. *p < 0.05, **p < 0.01, ****p < 0.0001, unpaired *t* test or one-way ANOVA. AM, alveolar macrophages; DC, dendritic cells; IM, interstitial macrophages; MPV, metapneumovirus.

PD-1 signaling regulates CD8^+^ T cell function in young HMPV-infected mice (12–15). In addition, HMPV-specific CD8^+^ T cells from aged mice demonstrated increased PD-1 expression (20). We hypothesized that PD-1/PD-L signaling also plays an important role in aged CD8^+^ T cell function during HMPV infection. To test this hypothesis, we first measured PD-1 and PD-L1/L2 expression on young and aged immune cells in the lung by analyzing publicly available scRNAseq datasets (8). *Pdcd1* was significantly increased on aged lung CD8^+^ T cells compared with young CD8^+^ T cells (Fig. 1A, 1B), whereas there was no difference in *Pdcd1* expression between age groups for CD4^+^ T cells or B cells (Fig. 1C, 1D). In our aged mouse HMPV model, aged mock-infected CD4^+^ T cells had increased *Pdcd1* expression compared with young CD4^+^ T cells, but there was no difference in CD19^+^ B cells (Supplemental Fig. 1A). We also found that upregulation of PD-1 during HMPV infection was specific to CD8^+^ T cells with no difference in PD-1 on CD4^+^ T or CD19^+^ B cells between young and aged HMPV-infected mice (Supplemental Fig 1B). PD-1 expression robustly increased with infection in young mice but stayed elevated in aged mice in both mock and HMPV infection (Fig. 1E). scRNAseq data revealed no difference in *Havcr2* (TIM-3), *Lag3*, or *Cd244* (2B4) inhibitory receptor expression on CD8^+^ T cells (Supplemental Fig. 1C–1E), which we confirmed at the protein level in mock-infected mice via flow cytometry (Supplemental Fig. 1F).

We performed a series of transplants and adoptive transfers of young or aged cells into young or aged hosts to test whether elevated PD-1 expression on aged CD8^+^ T cells is cell intrinsic or dependent on the host environment. A mixed BM chimera of congenically marked young or aged BM transplanted in a 1:1 ratio into lethally irradiated young or aged hosts revealed that there was a significant increase of PD-1 expression in aged CD8^+^ T cells compared with young CD8^+^ T cells in an aged host, with a similar trend for aged CD8^+^ T cells in a young host (Fig. 1F). When congenically marked bulk young or aged purified T cells were transplanted into lethally irradiated young or aged hosts, aged CD8^+^ T cells showed a trend toward increased PD-1 expression in a young host (Fig. 1G). There was no difference in PD-1 expression between aged and young CD8^+^ T cells in an aged host, but there was a significant increase in PD-1 in the aged CD8^+^ T cells in an aged host compared with young CD8^+^ T cells in a young host control (Fig. 1G). Last, to confirm if this phenomenon was specific to CD8^+^ T cells alone, we adoptively transferred young or aged CD8^+^ T cells along with young CD4^+^ T and B cells into *Rag1*^−/−^ recipients that lack all T and B lymphocytes. Aged CD8^+^ T cells significantly upregulated PD-1 in a young *Rag1*^−/−^ host compared with young CD8^+^ T cells (Fig. 1H). Overall, these findings indicate that there are both cell-extrinsic and cell-intrinsic effects impacting PD-1 expression on aged CD8^+^ T cells.

We next assessed expression of PD-L1 and PD-L2 in young versus aged mice. There were no significant differences in PD-L1 or PD-L2 expression between ages in mock- or HMPV-infected groups across numerous innate immune cell types (Fig. 1I and Supplemental Fig. 1G), with the exception of a small increase in PD-L2 expression in aged mock interstitial macrophages (Supplemental Fig. 1G). Notably, alveolar macrophages expressed very high levels of PD-L1 at baseline and under all conditions, as previously reported (33). We also found no difference between ages in *CD274* (PD-L1) (Fig. 1J) or in *PDCD1LG2* (PD-L2) (Supplemental Fig. 1H) in the lung myeloid cell scRNAseq dataset (8). Collectively, these data indicate that aged CD8^+^ T cells do exhibit a cell-intrinsic increase in PD-1 expression, but with a contribution from the host microenvironment.

### Proportion of granzyme B–expressing CD8^+^ T cells increases after PD-1 blockade

**FIGURE 2.**
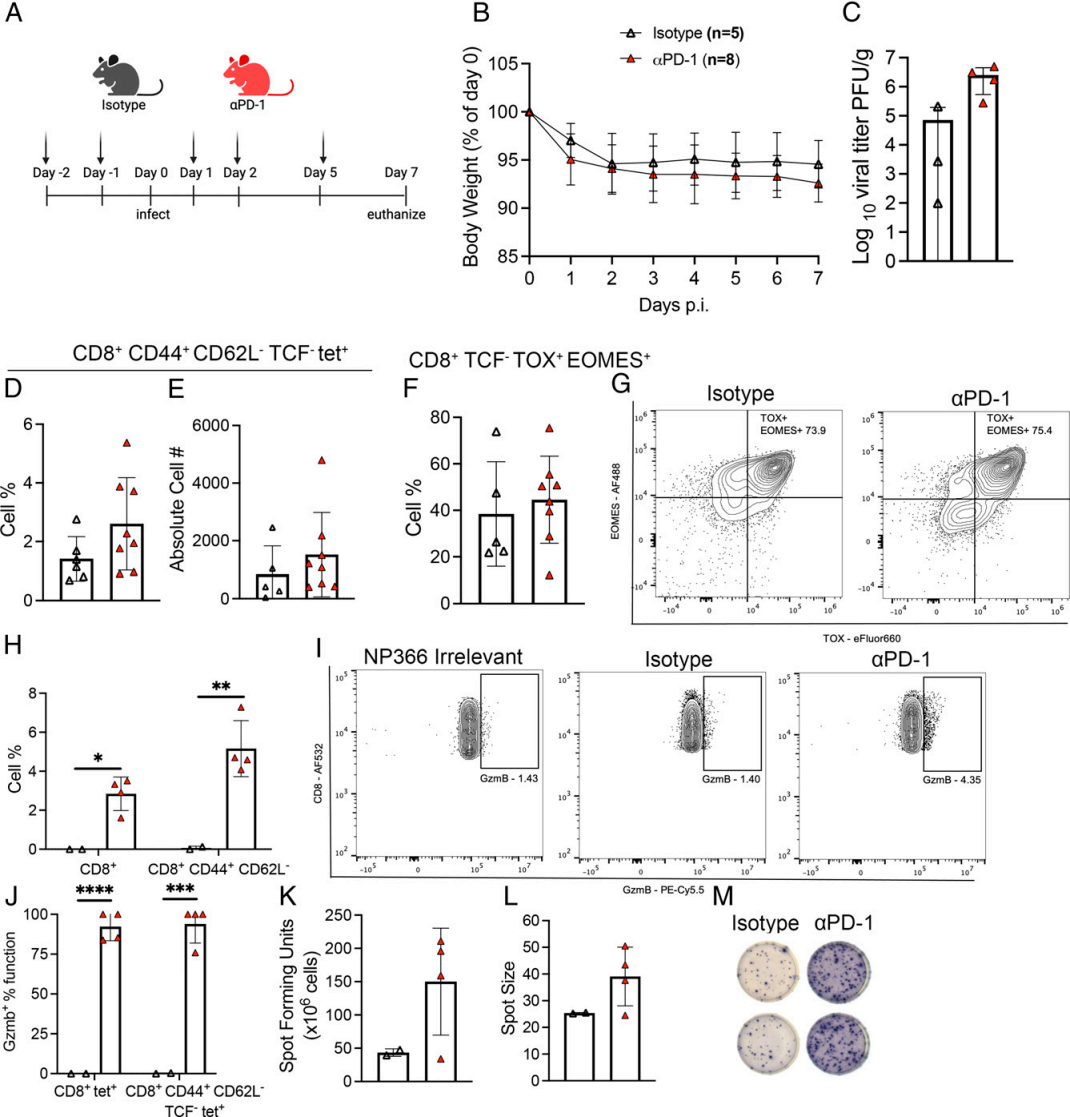
Proportion of granzyme B–expressing CD8^+^ T cells increases after PD-1 blockade. (**A**) Aged mice were treated with 200 g/200 l PD-1 or rat isotype control Ab via i.p. injection 2 d prior to infection and days 1, 2, and 5 p.i. (**B**) Weight loss during infection. (**C**) Viral titer in PFU/g between aged mice treated with isotype control of PD-1 blockade. (**D** and **E**) Cell percentage and absolute cell number of lung CD44^+^ CD62L^−^ TCF^−^ tet^+^ CD8^+^ T cells. (**F**) TCF^−^ TOX^+^ EOMES^+^ lung CD8^+^ T cells in aged mice treated with isotype or PD-1 blockade. (**G**) Representative flow plots of TOX and EOMES staining. (**H**) Bulk lung CD8^+^ and CD44^+^ CD62L^−^ Gzmb^+^ cell percentage. (**I**) Representative flow plots of Gzmb staining on lung CD8^+^ CD44^+^ CD62L^−^ T cells. (**J**) GzmB percentage function of lung CD8^+^ tet^+^ and CD8^+^ CD44^+^ CD62L^−^ TCF^−^ tet^+^ in both groups. (**K**) Spot number from ex vivo peptide stimulation IFN-γ ELISPOT of lung lymphocytes from aged isotype- or PD-1 blockade–treated mice and (**L**) spot size. (**M**) Representative images of ELISPOT wells. Absolute cell number calculation by BioLegend Precision Counting Beads. Each data point represents one individual mouse. Data in (B)–(G) represent three experimental replicates with two to four mice/group. Data in (H)–(J) represent two experimental replicates with two or three mice/group. Data in (K)–(M) represent one experimental replicate with two to four mice/group. *p < 0.05, **p < 0.01, ***p < 0.001, ****p < 0.0001.

Given the increased PD-1 expression on aged CD8^+^ T cells, we tested whether in vivo PD-1 blockade would rejuvenate TCF^−^ TOX^+^ EOMES^+^ exhausted (T_EX_) CD8^+^ T cells in aged mice. Studies have previously shown that PD-1 blockade improves CD8^+^ T cell function in chronic viral infection and cancer models (34, 35). We treated aged mice with isotype control or PD-1 blocking Ab on the two days prior to HMPV infection and days 1, 2, and 5 p.i. (Fig. 2A). We found no significant difference in weight loss (Fig. 2B) or viral burden in isotype control or anti–PD-1–treated mice (Fig. 2C). PD-1 blockade had no significant impact on the production of HMPV-specific lung CD8^+^ T cells, based on tetramer staining with the HMPV viral epitope H2-D/K N_11-18_ (N11) (Fig. 2D, 2E), and also did not impact the T_EX_ CD8^+^ T cells that expressed TOX and EOMES (Fig. 2F, 2G; Supplemental Fig. 1I–1K). However, in vivo PD-1 blockade in aged mice resulted in a striking increase in granzyme B production in CD44^+^ CD62L^−^ CD8^+^ T cells (Fig. 2H, 2I), which was even more pronounced for HMPV-specific (or N11 tetramer^+^) CD8^+^ CD44^+^ TCF^−^ cells (Fig. 2J). IFN-γ production did not significantly increase in aged mice treated with PD-1 blockade (Fig. 2K–M). Thus, PD-1 blockade alone appeared to have a favorable impact on HMPV-specific CD8^+^ T cell granzyme B production.

### Aged *Pdcd1*^−/−^ mice have improved CD8^+^ T cell granzyme B production during HMPV infection

**FIGURE 3.**
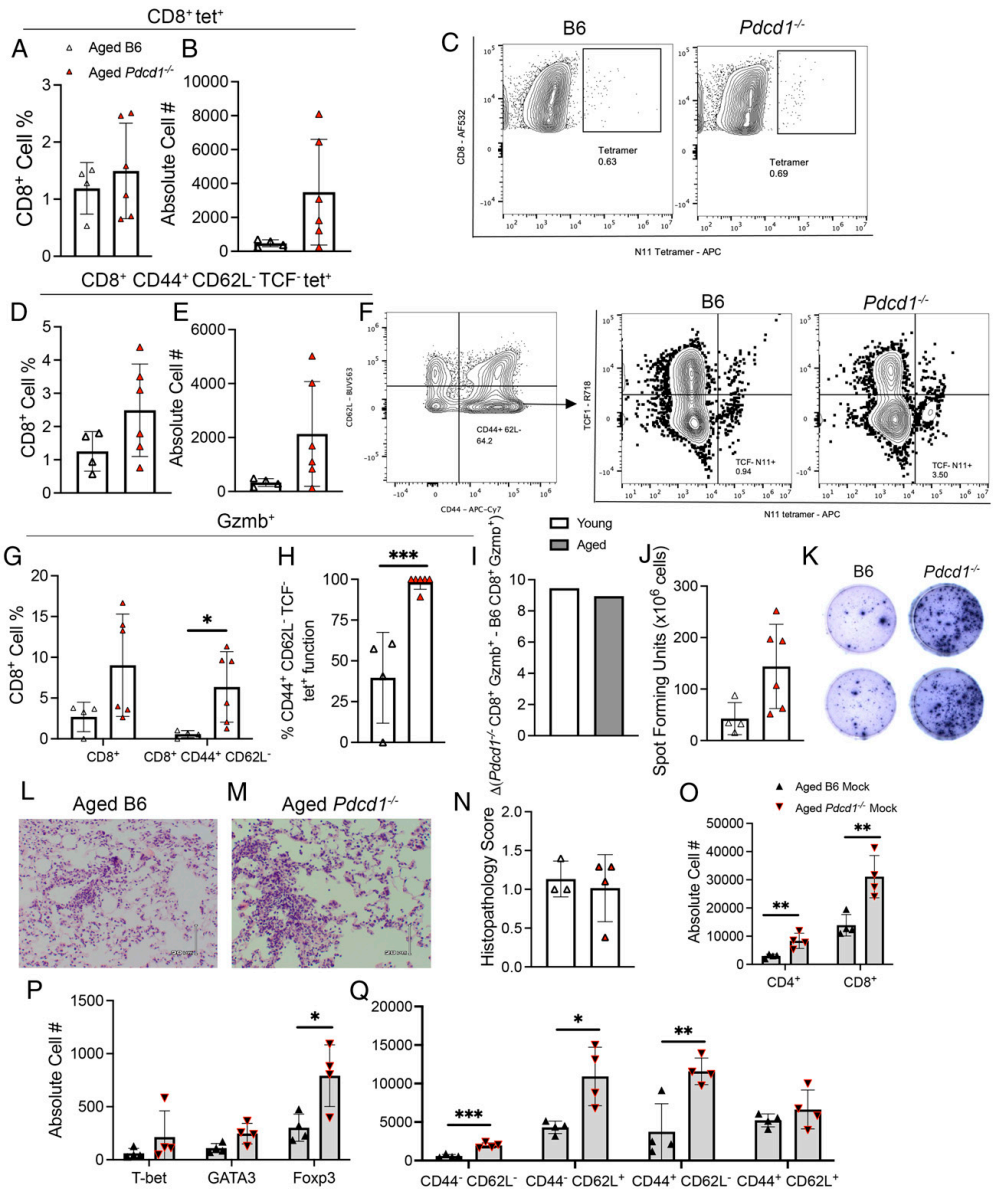
Aged *Pdcd1*^−/−^ mice have improved CD8^+^ T cell granzyme B production during HMPV infection. (**A** and **B**) Cell percentage (left) and absolute cell number (right) of lung CD8^+^ tet^+^ T cells in B6 and *Pdcd1*^−/−^ mice at day 7 p.i. (**C**) Representative flow plots of tetramer staining. (**D** and **E**) Cell percentage (left) and absolute cell number (right) of lung CD8^+^ CD44^+^ CD62L^−^ TCF^−^ tet^+^ cells in aged B6 and *Pdcd1*^−/−^ mice at day 7 p.i. (**F**) Representative flow plots of TCF^−^ tetramer staining. (**G**) Granzyme B^+^ bulk CD8^+^ and CD44^+^ CD62L^−^ CD8^+^ T cells. (**H**) Percentage function of granzyme B^+^ CD44^+^ CD62L^−^ TCF^−^ tet^+^ lung CD8^+^ T cells. (**I**) Δ Change of *Pdcd1*^−/−^ CD8^+^ Gzmb^+^ T cells minus B6 CD8^+^ Gzmb^+^ T cells in young and aged HMPV-infected mice at day 7 p.i. (**J**) ELISPOT ex vivo peptide stimulation IFN-γ production from B6 or *Pdcd1*^−/−^ lung lymphocytes. (**K**) Representative ELISPOT well images. (**L** and **M**) H&E images of aged B6 and *Pdcd1*^−/−^ lungs on day 7 p.i. (**N**) Histopathological scoring from both groups of mice. (**O**) Absolute cell number of CD4^+^ and CD8^+^ T lymphocytes, (**P**) T-bet, GATA3, Foxp3^+^ CD4^+^ T lymphocytes, and (**Q**) CD44 and CD62L expression on CD8^+^ T lymphocytes in aged B6 mice or *Pdcd1*^−/−^ mock-infected mice. Absolute cell number calculation by BioLegend Precision Counting Beads. The histopathological score is the average score per section field by a group-blinded experienced lung pathologist: 0, no inflammation; 1, <25% inflammation; 2, 25–50% inflammation; 3, 50–75% inflammation; and 4, >75% inflammation. Each data point represents one individual mouse. Data in (A)–(K) represent two experimental replicates with two or three mice/group. Data in (L) and (M) represent one experimental replicate with three or four mice/group. *p < 0.05, **p < 0.01, ***p < 0.001, unpaired *t* test or one-way ANOVA.

As a complementary approach to Ab blockade, we used *Pdcd1*^−/−^ mice, which exhibited increased CD8^+^ T cell function during HMPV infection in young 6–8-wk-old mice (12). We therefore infected aged *Pdcd1*^−/−^ mice and age-matched WT mice and evaluated the antiviral CD8^+^ T cell response at day 7 p.i. Of note, these age-matched *Pdcd1*^−/−^ and WT mice were not littermates. In addition, *Pdcd1*^−/−^ mice develop severe autoimmune disease by 9–10 mo, and thus these experiments used knockout mice that were at most 10 mo of age. We confirmed PD-1 expression was absent on *Pdcd1*^−/−^ CD8^+^ T cells (Supplemental Fig. 2A). Aged *Pdcd1*^−/−^ mice infected with HMPV had no difference in CD8^+^ N11-specific T cells at day 7 p.i. (Fig. 3A–3C) but exhibited a trend toward increased CD8^+^ CD44^+^ CD62L^−^ TCF^−^ N11-specific T cells (Fig. 3D–3F). There was no significant difference in weight loss (Supplemental Fig. 2B) or viral titer (Supplemental Fig. 2C) in aged mice. We have previously shown that blocking the PD-1 pathway in young mice improves viral clearance (12). No difference was seen in TIM-3 expression between aged *Pdcd1*^−/−^ and WT mice (Supplemental Fig. 2D, 2E). However, LAG-3 expression was increased (Supplemental Fig. 2F, 2G) on *Pdcd1*^−/−^ CD8^+^ tet^+^ T cells. PD-1 absence did not significantly affect the expression levels or absolute cell numbers of CD44 and CD62L on CD8^+^ T cells (Supplemental Fig. 2H, 2K). We also assessed whether the absence of PD-1 affected the terminal differentiation of CD4^+^ T cell subsets. We found no significant differences in the frequency or absolute cell number of CD4^+^ Foxp3^+^, T-bet^+^, GATA3^+^ (Supplemental Fig. 2I, 2L), or Th1/Th2 ratio between genotypes (Supplemental Fig. 2J). We also found no difference in accumulation of inflammatory infiltrates in the lung as measured by histopathological score (Fig. 3L–3N).

Considering the minimal differences in clinical disease, CD8^+^ tetramer production, and CD8^+^ differentiation to effector (CD44^+^ CD62L^−^) and memory (CD44^−^ CD62L^+^; CD44^+^ CD62L^+^) subsets, we next assessed CD8^+^ T cell antiviral function in the presence and absence of PD-1. There was a trend toward increased granzyme B expression in bulk *Pdcd1*^−/−^ CD8^+^ T cells and a significant increase in granzyme B production in CD44^+^ CD62^−^ CD8^+^ T cells (Fig. 3G). There was also a striking increase in CD44^+^ CD62L^+^ TCF^−^ tet^+^ granzyme B percentage function in aged *Pdcd1*^−/−^ mice (Fig. 3H).

We next assessed whether this increase in CD8^+^ T cell function was comparable to the increase in function we observed in young *Pdcd1*^−/−^ T cells compared with young WT T cells both in this study and in our previous studies (Supplemental Fig. 2M) (12–15). The δ change between *Pdcd1*^−/−^ and WT Gzmb^+^ CD8^+^ T cells for both age groups showed a similar increase in granzyme B production (Fig. 3I). This finding indicates that granzyme B production of CD8^+^ T cells is improved in the absence of PD-1 signaling to a similar degree, regardless of age. There was no significant increase in IFN-γ production in aged *Pdcd1*^−/−^ CD8^+^ T cells, indicating that PD-1 signaling effects were specific to granzyme B production (Fig. 3J, 3K).

We did investigate baseline characteristics of CD4^+^ and CD8^+^ T lymphocytes in mock-infected aged B6 and *Pdcd1*^−/−^ mice. Interestingly, aged *Pdcd1*^−/−^ mock-infected mice had increased absolute cell numbers of CD4^+^ and CD8^+^ T lymphocytes in the lung at baseline (Fig. 3O). Aged *Pdcd1*^−/−^ mice also had more CD4^+^ Foxp3^+^ T lymphocytes (Fig. 3P). Last, aged *Pdcd1*^−/−^ mice accumulated more CD44^−^ CD62L^−^, CD44^−^ CD62L^+^, and CD44^+^ CD62L^−^ CD8^+^ T lymphocytes at baseline (Fig. 3Q).

Overall, these results indicate that the absence of PD-1 in aged mice has a specific effect on improving granzyme B production in CD8^+^ T cells during HMPV infection but no discernible clinical effect on the outcome of viral infection.

### Aged *Pdcd1*^−/−^ influenza-infected mice also exhibited improved CD8^+^ T cell granzyme B production

**FIGURE 4.**
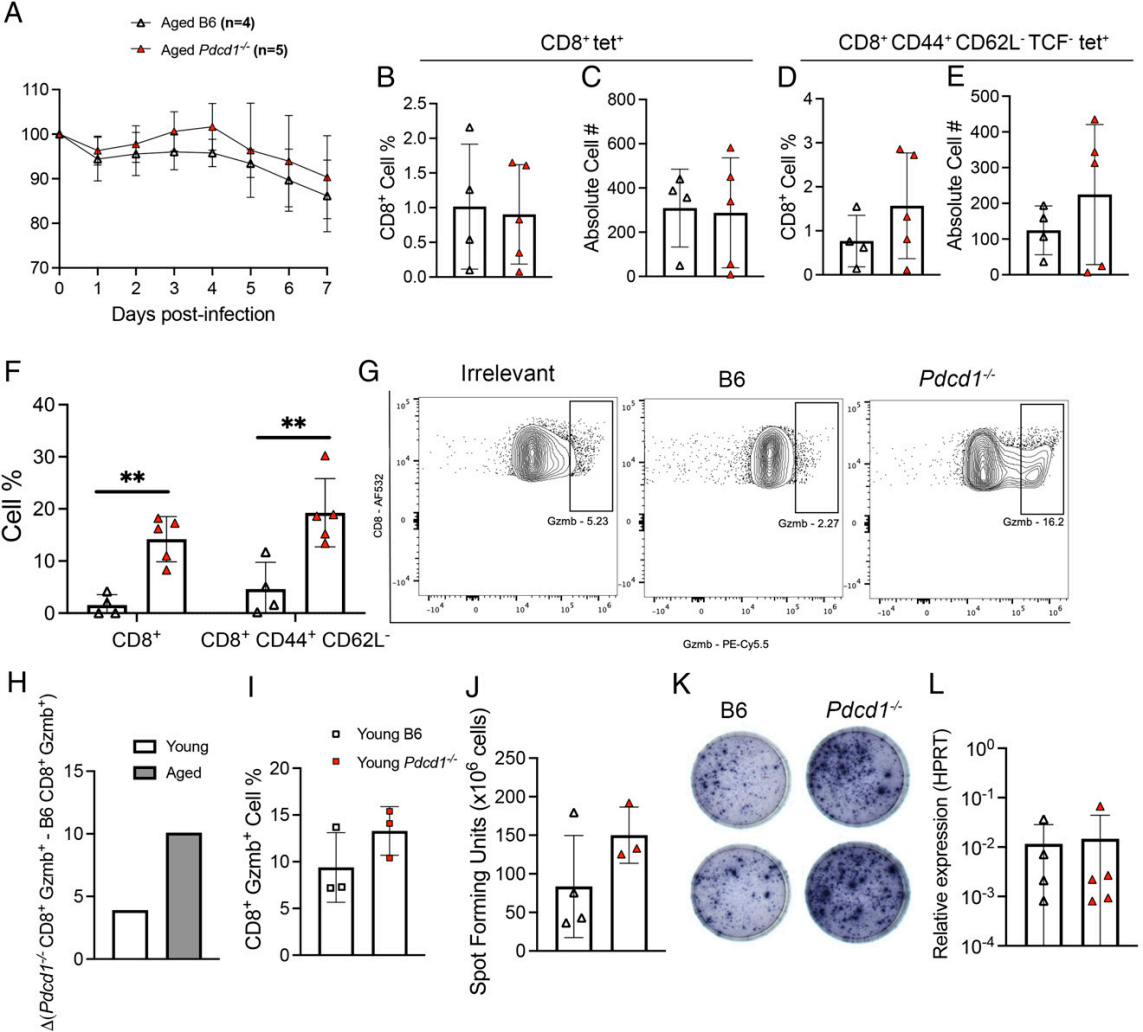
Aged *Pdcd1*^−/−^ influenza-infected mice also exhibited improved CD8^+^ T cell granzyme B production. (**A**) Weight loss of aged B6 and *Pdcd1*^−/−^ mice during influenza PR8 infection. (**B** and **C**) Bulk lung CD8^+^ tet^+^ cell percentage and absolute cell number. (**D** and **E**) Terminally differentiated lung CD8^+^ CD44^+^ CD62L^−^ TCF^−^ tet^+^ cell percentage and absolute cell number. (**F**) Bulk lung CD8^+^ T cell and CD8^+^ CD44^+^ CD62L^−^ granzyme B production. (**G**) Representative flow plots of granzyme B staining. (**H**) Δ Change of *Pdcd1*^−/−^ CD8^+^ Gzmb^+^ T cells minus B6 CD8^+^ Gzmb^+^ in lungs of young and aged PR8-infected mice. (**I**) Lung CD8^+^ granzyme B^+^ cell percentage in young B6 or *Pdcd1*^−/−^ mice. (**J**) Spot-forming units from IFN-γ ELISPOT ex vivo class I peptide stimulation from aged B6 and *Pdcd1*^−/−^ lung lymphocytes. (**K**) Representative images of wells from IFN-γ ELISPOT. (**L**) Quantitative RT-PCR relative expression to housekeeping gene (HPRT) or influenza A transcripts in lung homogenate. Absolute cell number calculation by BioLegend Precision Counting Beads. Each data point represents one individual mouse. Data (A)–(G) and (I) represent two experimental replicates with two or three mice/group. Data in (H) represent one experimental replicate with three mice per group. Data in (J)–(L) represent one experimental replicate with three or four mice/group. **p < 0.05, unpaired *t* test or two-way ANOVA.

To test whether these findings were generalizable to other respiratory viruses, aged B6 or *Pdcd1*^−/−^ mice were infected with influenza strain PR8 and euthanized on day 7 p.i. to assess the CD8^+^ T cell response. There was no significant difference in weight loss between the two groups (Fig. 4A). We again observed no difference in bulk CD8^+^ tet^+^ cell frequency or absolute cell number (Fig. 4B, 4C). There was also no difference in CD44^+^ CD62L^−^ TCF1^−^ tet^+^ T cells in *Pdcd1*^−/−^ mice (Fig. 4D, 4E). Similar to aged *Pdcd1*^−/−^ HMPV-infected mice, aged *Pdcd1*^−/−^ CD8^+^ T cells had significantly improved granzyme B production during PR8 influenza infection (Fig. 4F, 4G), with no significant difference in IFN-γ production (Fig. 4J, 4K). The δ change in granzyme B production (Fig. 4H) between *Pdcd1*^−/−^ and WT CD8^+^ T cells revealed that aged *Pdcd1*^−/−^ CD8^+^ T cells had a robust increase in granzyme B function, surpassing that of young *Pdcd1*^−/−^ CD8^+^ T cells (Fig. 4I). In addition, there was no difference in influenza titer in the lung between the two groups (Fig. 4L). These findings suggest that the absence of PD-1 in aged mice selectively improves CD8^+^ T granzyme B production similar to or exceeding that of young mice against multiple respiratory viruses.

### Aged *Pdcd1*^−/−^ CD8^+^ T cells transplanted into young mice had increased granzyme B production

**FIGURE 5.**
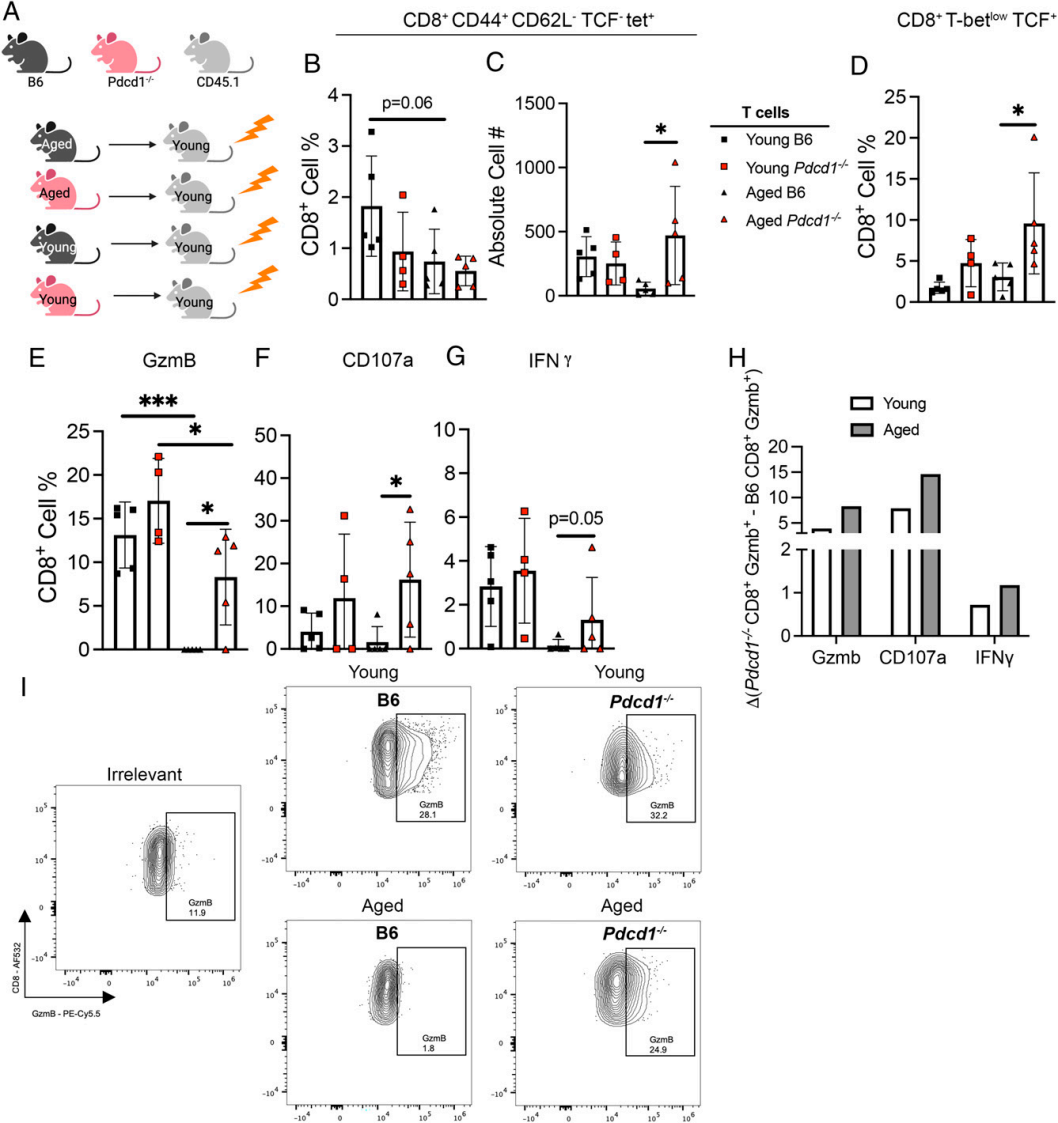
Aged *Pdcd1*^−/−^ CD8^+^ T cells transplanted into young mice had increased granzyme B production. (**A**) Experimental schematic. Aged or young B6 or *Pdcd1*^−/−^ T cells, young B6 CD19^+^ B cells, and young *Rag1*^−/−^ BM were transplanted into lethally irradiated CD45.1 young recipients. At 6 wk after transplant, mice were infected with HMPV, and CD8^+^ T cell response was assessed on day 7 p.i. (**B** and **C**) TCF^−^ tet^+^ lung CD8^+^ cell percentage and absolute cell number in all four groups. (**D**) Cell percentage of stem cell–like T-bet^low^ TCF^+^ lung CD8^+^ T cells. (**E**–**G**) Lung CD8^+^ T cells positive for granzyme B, CD107a, and IFN-γ, respectively. (**H**) Δ Change of donor *Pdcd1*^−/−^ CD8^+^ Gzmb^+^ T cells minus B6 CD8^+^ Gzmb^+^ T cells in young HMPV-infected recipient. (**I**) Representative flow plots of granzyme B staining. Each data point represents one individual mouse. Data represent one experimental replicate with four or five mice/group. *p < 0.05, ***p < 0.001, one-way ANOVA.

To test whether increased CD8^+^ T cell granzyme B production in the absence of PD-1 was cell intrinsic, young or aged WT or *Pdcd1*^−/−^ T cells were transplanted into lethally irradiated young CD45.1 mice along with young CD45.1 B cells and *Rag1*^−/−^ BM to reconstitute the myeloid compartment (Fig. 5A). At 6 wk after transplant, recipient mice were infected with HMPV and euthanized at day 7 p.i. We confirmed the identity of transplanted T cells by assessing PD-1 expression on CD45.2^+^ CD8^+^ T cells (Supplemental Fig. 3A). There was no difference in weight loss or viral burden between the four groups (Supplemental Fig. 3B, 3C). We also assessed engraftment of recipient (CD45.1) and donor (CD45.2) total CD3^+^ lymphocytes, CD4^+^ T cells, and CD8^+^ T cells (Supplemental Fig. 3D). *Pdcd1*^−/−^ lymphocytes engrafted better than B6 lymphocytes (Supplemental Fig. 3D–3G), especially in bulk CD3^+^ (Supplemental Fig. 3E) and CD8^+^ T lymphocytes (Supplemental Fig. 3G), as previously observed (15).

There were no significant differences in CD8^+^ CD44^+^ CD62L^−^ TCF^−^ tet^+^ cell percentage or absolute cell number between mice that received aged WT or aged *Pdcd1*^−/−^ T cells, with aged *Pdcd1*^−/−^ T cells producing more TCF^−^ tet^+^ CD8^+^ T cells (Fig. 5B, 5C). However, there was a significant increase in stem cell–like CD8^+^ T-bet^low^ TCF^+^ T cells previously described in (22) in aged *Pdcd1*^−/−^ compared with aged B6 mice, which could indicate a shift in the bulk CD8^+^ T cell repertoire toward accumulating more stem cell–like CD8^+^ T cells (Fig. 5D). Most notably, there was a significant increase in granzyme B production (Fig. 5E, 5I) and degranulation (CD107a^+^) (Fig. 5F), with a trend toward increased IFN-γ production (Fig. 5G), in aged *Pdcd1*^−/−^ CD8^+^ T cells compared with aged WT CD8^+^ T cells transplanted into young recipients. In addition, we assessed if the absence of PD-1 improved the function of aged CD8^+^ T cells relative to young CD8^+^ T cells. Aged *Pdcd1*^−/−^ CD8^+^ T cells exhibited improved function by all three measures to an extent similar to that of young *Pdcd1*^−/−^ compared with age-matched B6 CD8^+^ T cells (Fig. 5H).

To remove irradiation as a confounding variable, we performed an adoptive transfer of young or aged *Pdcd1*^−/−^ CD8^+^ T cells into *Rag1*^−/−^ young recipients along with young WT CD4^+^ T cells and B cells (Supplemental Fig. 4A). When we compared the function of young and aged *Pdcd1*^−/−^ CD8^+^ T cells in this model, we observed that young *Pdcd1*^−/−^ CD8^+^ T cells were still more functional in granzyme B and IFN-γ production than aged *Pdcd1*^−/−^ CD8^+^ T cells (Supplemental Fig. 4B–4D).

Taken together, these data indicate that the absence of PD-1 improves engraftment of not just CD8^+^ T cells but CD4^+^ T cells as well. We hypothesize that the increased granzyme B production in aged *Pdcd1*^−/−^ CD8^+^ T cells may be a result of a cell-intrinsic effect caused by the absence of PD-1 signaling or simply a difference in the homeostatic expansion based on the improved engraftment ability of *Pdcd1*^−/−^ lymphocytes.

### Aged *Pdcd1*^−/−^ had fewer T_RM_ cells 40 dpi

**FIGURE 6.**
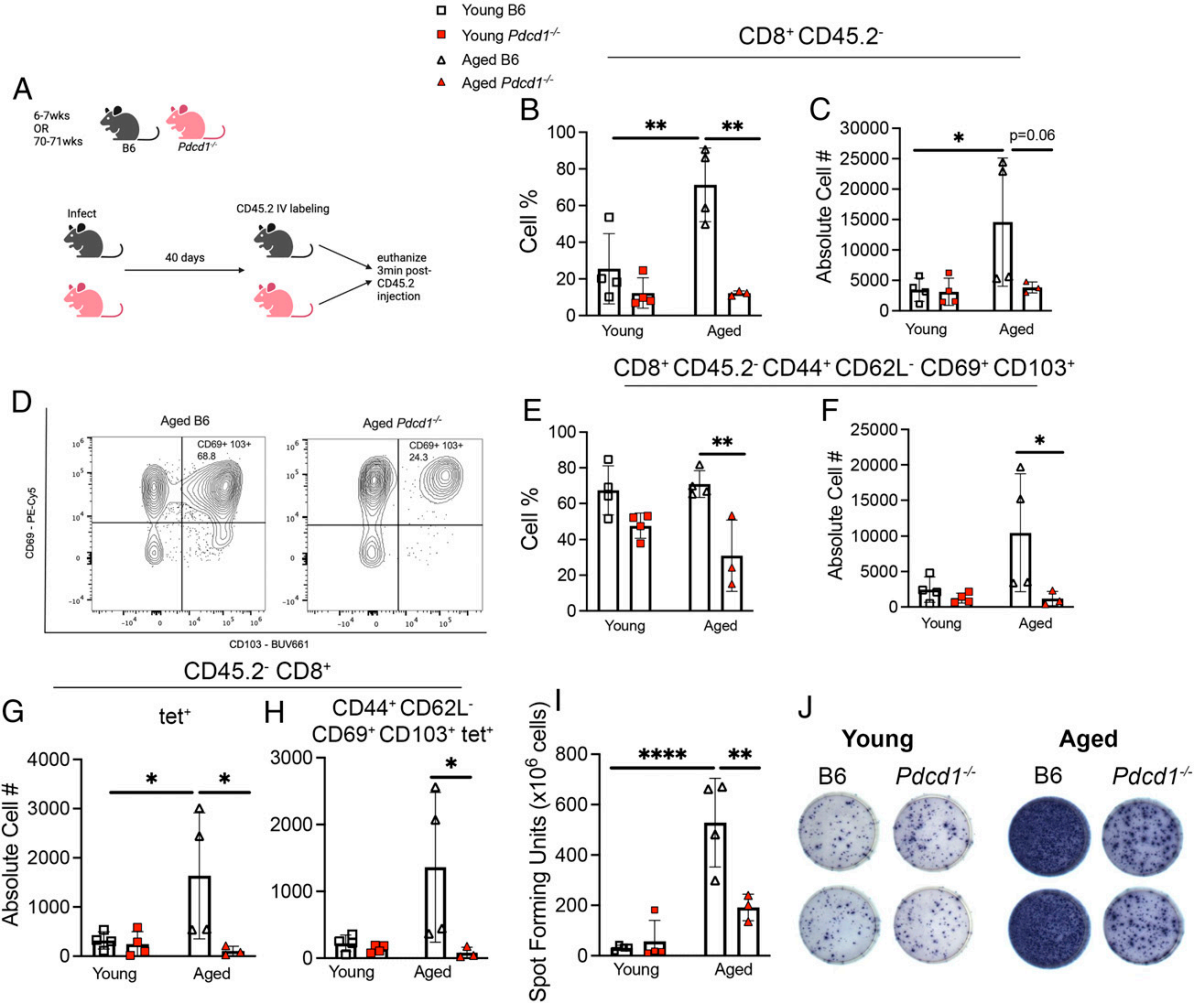
Aged *Pdcd1*^−/−^ had fewer T_RM_ cells at 40 d p.i. (**A**) Experimental schematic. (**B** and **C**) CD8^+^ CD45.2^−^ cell percentage and absolute cell number in lungs of young and aged B6 and *Pdcd1*^−/−^ mice. (**D**) Representative flow plots of CD69 and CD103 staining. (**E** and **F**) Cell percentage and absolute cell number of CD8^+^ CD45.2^−^ CD44^+^ CD62L^−^ CD69^+^ CD103^+^ in all four groups. (**G** and **H**) Absolute cell number of bulk lung CD45.2^−^ CD8^+^ tet^+^ and memory CD44^+^ CD62L^−^ CD69^+^ CD103^+^ T cells in all four groups. (**I**) Spot-forming units from IFN-γ ELISPOT ex vivo class I peptide stimulation from lung lymphocytes. (**J**) Representative images from IFN-γ ELISPOT wells. Absolute cell number calculated using BioLegend Precision Counting Beads. Each data point represents one individual mouse. Data represent one experimental replicate with three or four mice/group. *p < 0.05, **p < 0.01, ****p < 0.0001, one-way ANOVA.

Because nearly all children are seropositive for HMPV by age 5 y, adult infections represent reinfections (1). Although adult serum Ab titer affects susceptibility to HMPV reinfection and disease (36), little is known about T cell memory and adult HMPV reinfection. Thus, we tested how PD-1 signaling affected CD8^+^ T cell memory formation in young and aged mice. At day 40 p.i., aged or young B6 or *Pdcd1*^−/−^ mice were injected with CD45.2 Ab to label blood (CD45^+^) versus tissue (CD45^−^) cells and then immediately euthanized (Fig. 6A). Young WT and *Pdcd1*^−/−^ mice were included in this study to assess whether any differences we observed in the aged *Pdcd1*^−/−^ was due to the absence of PD-1 signaling, regardless of age. As shown previously (21), aged B6 mice accumulated significantly more CD8^+^ CD45.2^−^ T cells in the lungs than young B6 mice (Fig. 6B, 6C). However, aged *Pdcd1*^−/−^ mice had significantly fewer lung CD8^+^ CD45.2^−^ T cells than aged WT B6 mice (Fig. 6B, 6C). This decrease in CD8^+^ CD45.2^−^ T cells was specific to aged mice because there was no similar difference observed in young mice (Fig. 6B, 6C). In addition, aged *Pdcd1*^−/−^ mice also had significantly fewer lung CD8^+^ CD45.2^−^ CD44^+^ CD62L^−^ CD69^+^ CD103^+^ T_RM_ cells (Fig. 6D–6F) than aged WT mice in both cell percentage (Fig. 6E) and absolute cell number (Fig. 6F). There was no significant difference in CD8^+^ CD45.2^−^ CD44^+^ CD62L^−^ CD69^+^ CD103^+^ T_RM_ cell production in young mice of either genotype (Fig. 6E, 6F), indicating that this difference in CD8^+^ T cell memory formation was specific to aged mice. Aged *Pdcd1*^−/−^ mice also produced fewer lung HMPV-specific CD8^+^ CD45.2^+^ N11 tet^+^ and CD44^+^ CD62L^−^ CD69^+^ CD103^+^ N11 tet^+^ T cells than aged WT mice (Fig. 6G, 6H). Last, there was minimal IFN-γ production in young WT and *Pdcd1*^−/−^ mice at day 40 p.i. (Fig. 6I, 6J). However, there was robust IFN-γ production from aged WT T cells, as previously shown (21), which was significantly diminished in *Pdcd1*^−/−^ aged mice (Fig. 6I, 6J). These findings suggest that there is an age-associated, PD-1–dependent increase in lung tissue T_RM_ CD8^+^ cells.

### PD-1 blockade improves weight loss during reinfection of aged mice

**FIGURE 7.**
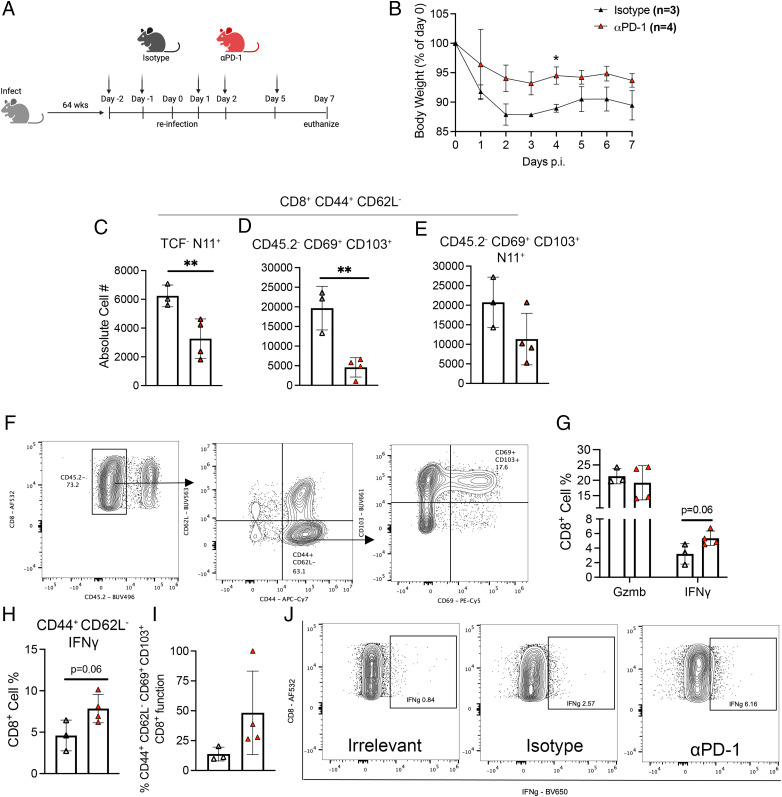
PD-1 blockade improves weight loss and memory CD8^+^ T cell function during reinfection of aged mice. (**A**) Experimental schematic. (**B**) Weight loss after rechallenge. (**C**–**E**) Absolute cell number of lung CD8^+^ CD44^+^ CD62L^−^. (**F**) Representative flow plots of CD69 and CD103 staining. (**G**) Lung CD8^+^ Gzmb^+^ and IFN-γ^+^ cell percentage in both groups. (**H**) CD8^+^ CD44^+^ CD62L^−^ IFN-γ^+^ cell percentage in lung. (**I**) Memory CD8^+^ T cell IFN-γ percentage function in lung. (**J**) Representative flow plots of IFN-γ staining. Absolute cell number calculation by BioLegend Precision Counting Beads. Each data point represents one individual mouse. Data represent one experimental replicate with three or four mice/group. *p < 0.05, **p < 0.01, unpaired *t* test or two-way ANOVA.

To model reinfection of aged adults, B6 mice were infected with HMPV at 6–7 wk, aged in-house, and rechallenged 14 mo p.i. (21). Two days prior to rechallenge and on days 1, 2, and 5 following rechallenge, aged mice were treated with either PD-1 blockade or isotype control (Fig. 7A). PD-1 blockade prevented weight loss in rechallenged aged mice compared with isotype treated control animals (Fig. 7B), but both groups had no detectable viral titer by day 7 after rechallenge (Supplemental. Fig. 4E). Viral replication may have been limited in this rechallenge by anti-HMPV Ab responses in all groups (Supplemental Fig. 4F). Aged αPD-1 mice produced fewer terminally differentiated TCF^−^ tet^+^ CD8^+^ T cells (Fig. 7C), bulk CD45.2^−^ CD69^+^ CD103^+^ (T_RM_) CD8^+^ T cells (Fig. 7D), and tetramer-specific CD45.2^−^ T_RM_ cells (Fig. 7E). Representative flow plots of T_RM_ gating are shown in Fig. 7F. Despite this decrease in production of T_RM_ cells, PD-1 blockade in aged mice on slightly increased bulk IFN-γ CD8^+^ T cell function (Fig. 7G, 7J) and IFN-γ production in CD44^+^ CD62L^−^ CD8^+^ T cells (Fig. 7H). In addition, the percentage function of IFN-γ-producing T_RM_ cells also tended to increase with PD-1 blockade (Fig. 7I). Overall, these data suggest that PD-1 blockade does impact CD8^+^ T_RM_ memory T cell production and weight loss in aged mice after rechallenge.

## Discussion

In this study, we investigated the role of PD-1 signaling in an aged mouse HMPV model. We found that (1) PD-1 expression was increased on aged CD8^+^ T cells (2), PD-1 signaling significantly impaired granzyme B production of aged CD8^+^ T cells against two respiratory viruses, (3) this improved function was primarily cell intrinsic or due to homeostatic differences in *Pdcd1*^−/−^ CD8^+^ T cells because aged B6 CD8^+^ T cells failed to improve cytokine production when transplanted into a young host, and (4) removal of PD-1 signaling resulted in accumulation of fewer cytotoxic CD8^+^ T_RM_ cells in aged mice.

We found that PD-1 was upregulated in aged mice in publicly available scRNAseq datasets (8). Our data in mock-infected aged mice corroborated these single-cell findings and further showed that PD-1 expression persisted on aged CD8^+^ T cells during HMPV infection, despite transplant into a young host. We have also further shown that PD-1 expression is increased on aged CD8^+^ HMPV-specific tetramer^+^ T cells during infection (20). In our mixed BM chimera transplant model, we observed this same phenomenon during HMPV infection. However, there was no difference in PD-1 expression on young or aged CD8^+^ T cells in an aged host in the bulk T cell syngeneic transplant model. Aging is multifactorial, and the aged microenvironment and well-defined inflammaging hypothesis (6, 7), as well as epigenetic changes in aged CD4^+^ T cells (10), could be possible explanations for these findings. For example, studies have identified an accumulation of inflammatory monocytes in aged mice (37, 38), which could propagate this inflammaging phenotype. Our own scRNAseq analysis of whole-lung cells from young and aged HMPV-infected mice corroborate published data (37, 38) identifying a unique inflammatory monocyte population present in uninfected aged mice that significantly expands only in aged mice following HMPV infection (O.B. Parks, T. Eddens, A. Evans, A. Kalavacharla, A. Adebiyi, and J.V. Williams, unpublished data). We hypothesize that these age-related changes in other immune cells significantly impact PD-1 expression on CD8^+^ T cells in the aged host, which could explain the findings in Fig. 1. Future investigations will elucidate the contribution of the aged microenvironment and somatic cells on CD8^+^ T cell function and epigenetic changes associated with increased age.

PD-1 blockade did not affect the population of T_EX_ CD8^+^ T cells that expressed TOX and EOMES, which are markers of terminal exhaustion (8, 10). This suggests that PD-1 signaling does not propagate or affect the expression of these markers in aged CD8^+^ T cells. This finding is consistent with reports that CD8^+^ T cell exhaustion can occur in the genetic absence of PD-1 (39); in that context, PD-1 signaling promoted excessive expansion and differentiation of cytotoxic CD8^+^ T cells (39), leading to more exhausted CD8^+^ T cells. In our model, *Pdcd1*^−/−^ aged mice tended to accumulate terminally differentiated CD8^+^ tet^+^ T cells. Importantly, at baseline, aged *Pdcd1*^−/−^ mice did have increased bulk CD4^+^ and CD8^+^ T lymphocytes with an increase in CD4^+^ Foxp3^+^ T lymphocytes and an increase in naive (CD44^−^ CD62L^+^) and effector (CD44^+^ CD62L^−^) CD8^+^ T lymphocytes. This does indicate that there are baseline differences between B6 and *Pdcd1*^−/−^ aged mice that could contribute to how aged *Pdcd1*^−/−^ mice respond to HMPV infection.

In addition, the absence of PD-1 signaling did not have an impact on the clinical outcome in aged mice (i.e., weight loss and viral titer). We hypothesize that this is due to the multifactorial nature of the immune response to HMPV, especially in the aged host. We have previously shown that CD8^+^ T cells are not the only cell type responsible for viral clearance, because CD8^+^ depletion in either aged or young mice had minimal effect on weight loss or viral clearance during HMPV infection (20). Another study also supports these findings, indicating the dual role of both CD4^+^ and CD8^+^ T cells in affecting the clinical outcome in mice infected with respiratory viruses (40).

Notably, the absence of PD-1 signaling had a predominant effect on CD8^+^ T cell granzyme B production. It is well known that PD-1 blockade plays an important role in activation of CD8^+^ effector T cells during acute viral infection (41). We have previously shown that CD8^+^ T cell migration to the site of infection in the lung is not impaired (20). Because CD8^+^ T cell antiviral function occurs at the site of infection in the lung, we sought to examine the effects of PD-1 signaling on aged CD8^+^ T cell function in the lung. In the present study in both HMPV and influenza infection, aged *Pdcd1*^−/−^ CD8^+^ T cells exhibited increased granzyme B production, which was recapitulated in the transplant experiments. However, the impaired granzyme B production still present in the adoptive transfer of aged *Pdcd1*^−/−^CD8^+^ T cells compared with young *Pdcd1*^−/−^ CD8^+^ T cells indicates that aged CD8^+^ T cells have other age-related cell-intrinsic deficits that inhibit CD8^+^ T cell antiviral function (20). These findings also suggest that aged CD8^+^ T cells may require help from CD4^+^ T cells and B cells that are unrestrained by PD-1 signaling. Furthermore, blockade of other inhibitory receptors, including TIM-3, failed to improve CD8^+^ T cell function during HMPV infection to the same degree as PD-1 blockade (15). LAG-3 blockade has been shown to enhance CD8^+^ T cell function against HMPV, but also enhanced lung pathology, indicating that LAG-3 may play a role in ameliorating lung damage during infection (15).

Importantly, Ab blockade and *Pdcd1*^−/−^ mouse approaches globally block or remove PD-1 signaling from all cells. Thus, we cannot fully exclude the effect of other cells. PD-1 is known to play an important role in the function of CD4^+^ T lymphocytes, specifically Foxp3^+^ CD4^+^ regulatory T cells (42, 43). We saw some differences at baseline in the expression of CD4^+^ T cell transcription factors with increased absolute cell number of *Foxp3*^+^ CD4^+^ T cells. This indicates that, at least at the transcription level, removal of PD-1 signaling does have an effect on CD4^+^ T cell differentiation, which could contribute to our findings in this study. We previously showed that regulatory T cells play an integral role early during HMPV infection (44). However, we focused the present studies on PD-1 effects on CD8^+^ T cells for a number of reasons, including the preferential increase in PD-1 expression on aged CD8^+^ T cells but not CD4^+^ T cells; the central role of CD8^+^ T cells against HMPV (12–15); and cell-intrinsic, age-associated CD8^+^ T cell functional impairment (20).

Aged *Pdcd1*^−/−^ CD8^+^ T cells had improved granzyme B production compared with aged B6 CD8^+^ T cells even when transplanted into a young host. We previously showed in a transplant model that aged B6 CD8^+^ T cells exhibited cell-intrinsic impairment of granzyme B production that was not restored by a young microenvironment (20). In this study, we further show that in the absence of PD-1, aged *Pdcd1*^−/−^ CD8^+^ T cells have a cell-intrinsic increase in granzyme B production. The significant increase in engraftment of *Pdcd1*^−/−^ lymphocytes was also observed in a prior mixed BM chimera model (15). Others have found that the absence of PD-1 can cause an increase in activation and proliferation of T lymphocytes (28, 39), which could provide one explanation for this finding. We report the absolute cell numbers for these transplant results to try to account for the differences in engraftment. However, considering these findings, we cannot rule out the possibility that the improved granzyme B production in aged *Pdcd1*^−/−^ CD8^+^ T cells could be due to homeostatic or cell-intrinsic differences. Future studies can analyze this further by investigating the proliferation capacity and metabolism potential of *Pdcd1*^−/−^ CD8^+^ T cells. This increase in engraftment coupled with the increase in activation and terminal differentiation of *Pdcd1*^−/−^ CD8^+^ T cells could also present possible avenues for PD-1 blockade therapy to be used in conjunction with chimeric Ag receptor T cells. Furthermore, studies have found a synergistic effect in mice against tumors when chimeric Ag receptor T cells are genetically modified to express PD-1 blocking Abs (45, 46).

It is not fully clear why older adults are more likely to experience severe respiratory disease upon HMPV reinfection. HMPV and respiratory syncytial virus reinfections in aged humans and mice occur despite the presence of neutralizing Abs (36, 47, 48). Importantly, mice are semipermissive hosts for HMPV and thus are a limited model for reinfection. These findings together could explain why aged mice had an undetectable viral titer at day 7 after rechallenge. Our previous studies suggest that aged CD8^+^ T cell dysfunction contributes to severe HMPV disease in older individuals (20, 21). We and others have also previously found that CD8^+^ T_RM_ cells accumulate in aged mice and can contribute to increased disease severity and lung pathogenesis in HMPV (21) and influenza (49). This CD8^+^ T_RM_ accumulation was impaired in aged *Pdcd1*^−/−^ mice, which leads to the hypothesis that PD-1 signaling may be impacting the formation and accumulation of this CD8^+^ memory T cell population in the aged host. PD-1 signaling is required for optimal CD8^+^ memory T cell formation (28, 50). These studies reported that the timing of PD-1 blockade was important to optimize effector CD8^+^ T cell function without severely limiting memory formation (28, 41). Further supporting this, one study found that PD-1 signaling serves as a mediator to limit CD8^+^ T_RM_ activity in the lung during influenza infection, which helped prevent lung fibrosis (51). Because CD8^+^ T cells develop age-associated functional impairment and accumulate cytotoxic memory populations that contribute to severe disease in older adults infected with respiratory viruses (17–19), this opens up the possibility of using αPD-1 therapy in elderly individuals during a window of time shortly after they are infected with a respiratory virus.

Mice receiving PD-1 blockade upon rechallenge 14 mo after primary infection were clinically better (i.e., less weight loss), had fewer CD8^+^ T_RM_ cells, but had borderline more function. We suspect that PD-1 blockade performed on aged in-house mice 14 mo after primary infection was semieffective because the cytotoxic CD8^+^ T_RM_ population may have already formed. Therefore, PD-1 blockade 2 d prior to rechallenge and subsequent boosting postrechallenge may not have been sufficient to affect the CD8^+^ T_RM_ function to the same degree as our other models in this study. *Pdcd1*^−/−^ mice have never expressed PD-1, which may have a profound effect on CD8^+^ T_RM_ formation that is not observed when giving PD-1 blockade to WT aged mice. However, we are actively investigating the mechanism behind PD-1 blockade in 14 mo rechallenged mice. Although CD8^+^ T cells contribute to pathogenesis, other immune cells may have been affected by PD-1 blockade, leading to diminished inflammation and thus reduced weight loss. Future experiments and conditional knockout mice are needed to define the role of PD-1 on select immune cell subsets.

Taken together, in this study, we identified preferentially increased PD-1 expression on aged CD8^+^ T cells that persisted even when aged CD8^+^ T cells were transplanted into a young host. We also show that removal of PD-1 signaling has a positive effect on aged CD8^+^ T cell granzyme B production during multiple respiratory viral infections. We further demonstrated that *Pdcd1*^−/−^ aged mice did not develop a cytotoxic CD8^+^ T_RM_ population in the lung 40 d p.i. These results indicate a temporal role of PD-1 signaling in both the initial antiviral response to improve granzyme B production and during the resolution phase of infection, when the memory CD8^+^ T cell compartment is formed. Last, using an aged rechallenge model, we show that aged mice receiving PD-1 blockade lost less weight and produced fewer CD8^+^ T_RM_ cells. These findings can inform future studies interrogating the therapeutic potential of PD-1 blockade in aged humans infected with respiratory viruses.

## Supplementary Material

Supplemental Figures 1 (PDF)
