# Supplementary material for: PD-1 Impairs CD8+ T Cell Granzyme B Production in Aged Mice during Acute Viral Respiratory Infection
Source: Immunohorizons. 2023 Nov 28;7(11):771–87. doi: 10.4049/immunohorizons.2300094 (PMC10696419; doi:10.4049/immunohorizons.2300094)
Supplement: Supplemental Figures 1 (PDF) [file IH_2300094_Supplemental_1.pdf]

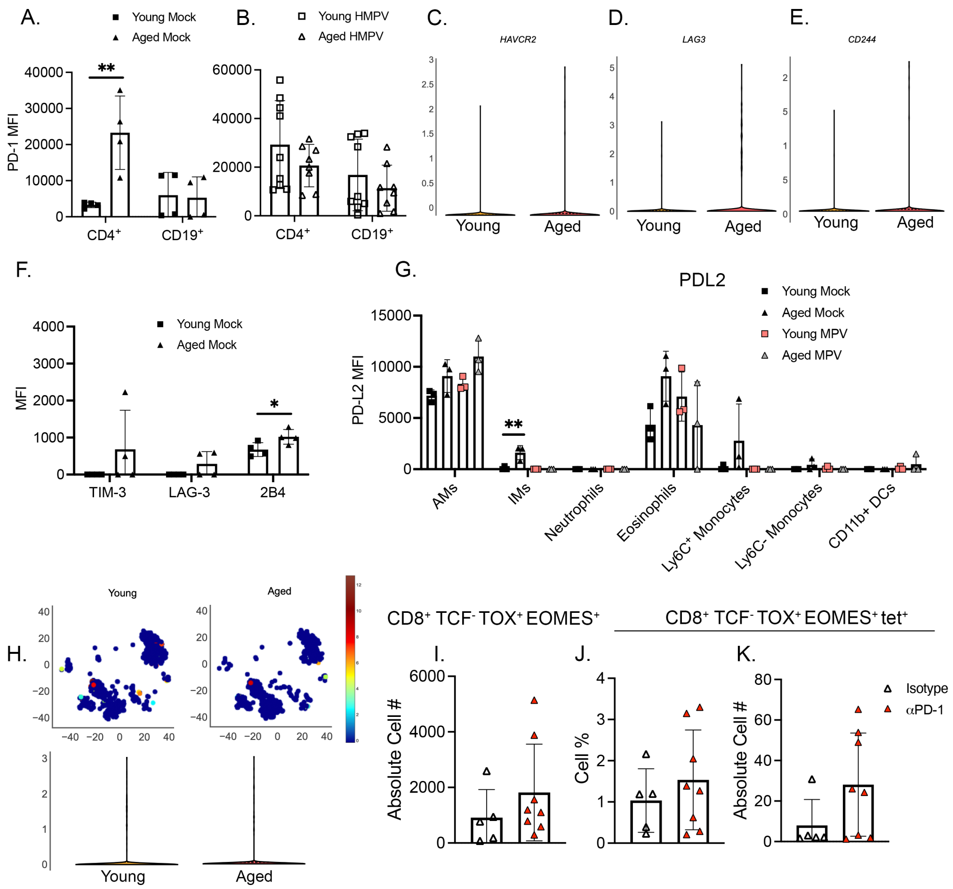

Supplemental 1. Minimal TIM-3, LAG-3, and 2B4 expression on aged CD8⁺ T cells and impaired tetramer production in aged mice treated with 4-1BB co-stimulation.

(A) PD-1 mean fluorescence intensity (MFI) on CD8$^+$ T cells in young and aged mock infected mice at day 7 p.i. **(B)** PD-1 MFI on CD8$^+$ T cells in young and aged HMPV infected mice at day 7 p.i. **(C-E)** Violin plots of *HAVCR2* (TIM-3), *LAG-3*, and *CD244* (2B4) expression from scRNAseq lung CD8$^+$ T cells in young and aged uninfected mice. **(F)** MFI of TIM-3, LAG-3, and 2B4 expression on lung CD8$^+$ T cells in young and aged mock infected mice. **(G)** PDL2 mean fluorescence intensity (MFI) on innate immune cells at day 1 p.i. **(H)** Heat map of *Pdcd1lg2* (PD-L2) expression in lung myeloid cells (TOP) and corresponding violin plot (BOTTOM). **(I-K)** Absolute cell number of CD8$^+$ TCF$^-$ TOX$^+$ EOMES$^+$ **(I)** or tetramer$^+$ cell percent **(J)** and absolute cell number **(K)** in isotype or PD-1 blockade treated aged HMPV-infected mice. Absolute cell number calculation by Biolegend Precision Counting Beads. *P<0.05; **P<0.01; unpaired t-test or two-way ANOVA. Data in (A; F) represents four experimental replicates, 1 mouse/group. Data in (B) represents four experimental replicates, 2-3 mice/group. Data in (G) represents one experimental replicate, 3 mice/group. Data in (I-K) represents three experimental replicates 3-4 mice/group.

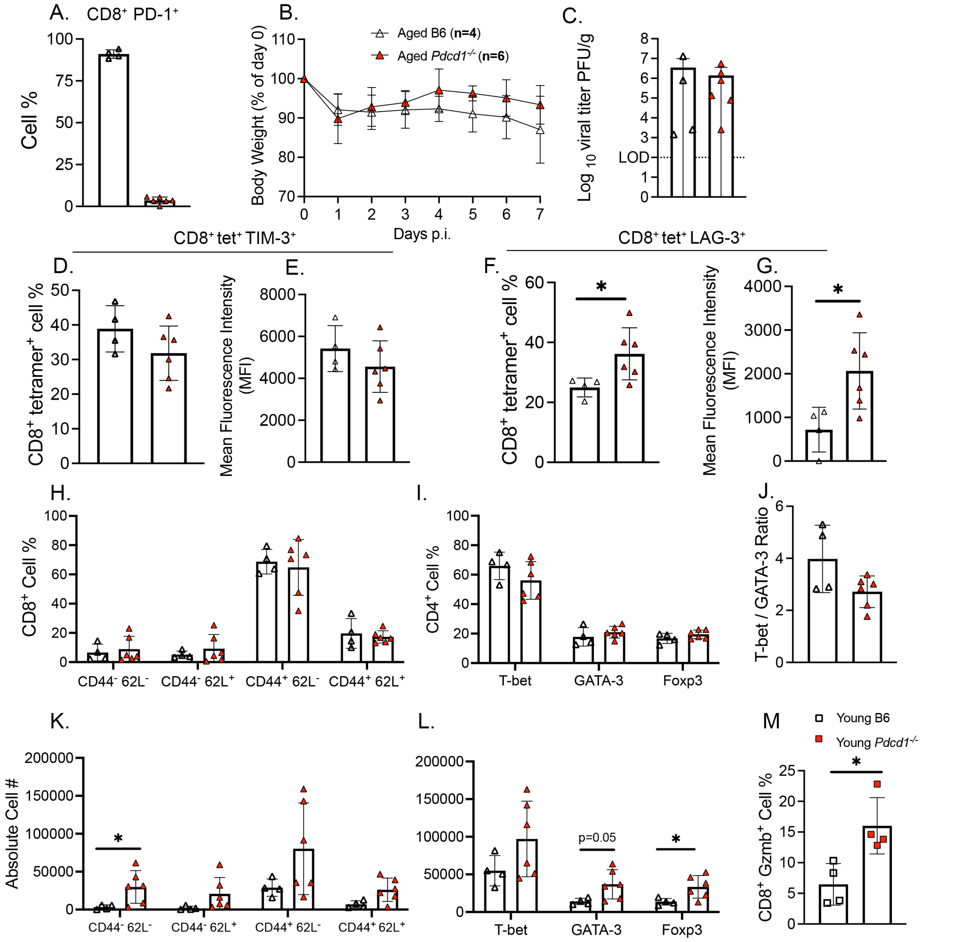

Supplemental Figure 2. Aged *Pdcd1*$^{-/-}$ tet^{+} CD8^{+} T cells had a compensatory increase in LAG-3 expression.
(A) PD-1 expression on CD8^{+} tet^{+} T cells in both B6 and *Pdcd1*$^{-/-}$ groups. **(B-C)** Weight loss and viral burden, respectively between aged B6 and *Pdcd1*$^{-/-}$. **(D-E)** TIM-3 expression in cell percent and MFI on CD8^{+} tet^{+} T cells. **(F-G)** LAG-3 expression in cell percent and MFI on CD8^{+} tet^{+} T cells. **(H & K)** CD44 and 62L expression on CD8^{+} T cells in aged B6 and *Pdcd1*$^{-/-}$.mice - cell percent and absolute cell number, respectively. **(I & L)** *T-bet, Foxp3, T-bet,* and *GATA3* expression on CD4^{+} T cells - cell percent and absolute cell number, respectively. **(J)** Th1:Th2 ratio between aged B6 and *Pdcd1*$^{-/-}$ mice. **(M)** CD8^{+} Gzmb^{+} cell percent in young B6 and *Pdcd1*$^{-/-}$ HMPV-infected mice. *P<0.05; unpaired t-test. Data represents two experimental replicates, 2-3 mice/group.

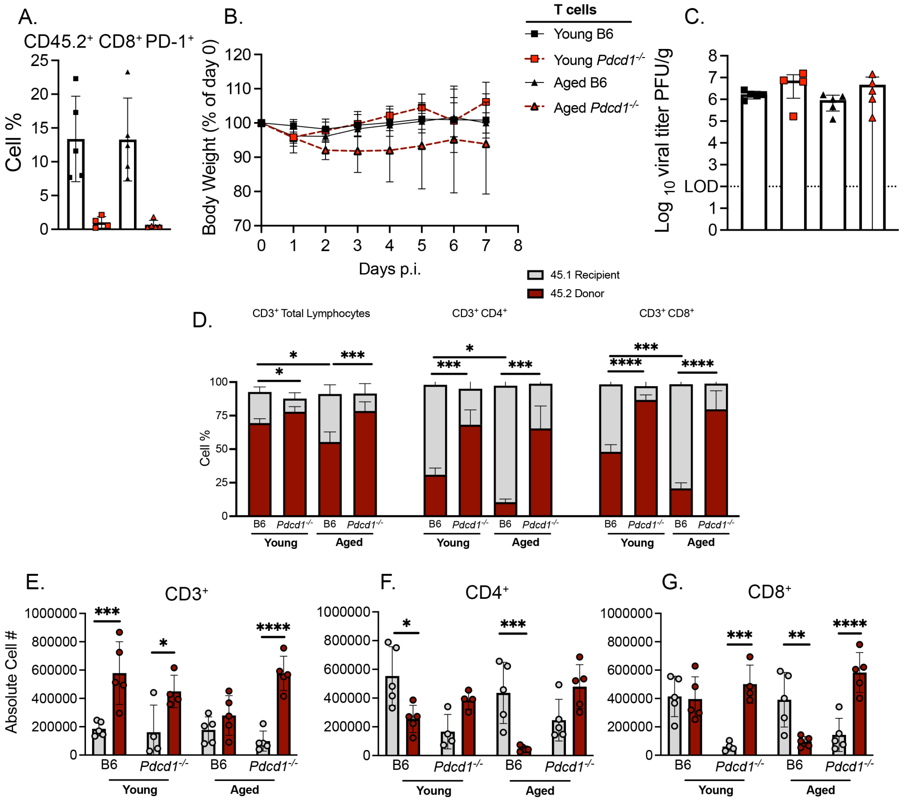

Supplemental Figure 3. Aged and young *Pdcd1$^{-/-}$* T cells had better engraftment in syngeneic transplant. **(A)** PD-1 expression on donor transplanted CD8$^+$ T cells. **(B)** Weight loss in the four syngeneic transplant groups during HMPV infection. **(C)** Viral titer measured in PFU/g at day 7 p.i. **(D)** Cell frequency of CD45.1 recipient (gray bars) and CD45.2 donor (red bars) in CD3$^+$ total lymphocytes, CD3$^+$ CD4$^+$, and CD3$^+$ CD8$^+$ T lymphocytes. **(E-G)** Absolute cell number of CD45.1 and CD45.2 CD3$^+$, CD4$^+$, and CD8$^+$ T cells. *P<0.05; **P<0.01, ***P<0.001; ****P<0.0001, unpaired t-test or one-way ANOVA. Absolute cell number calculated by BioLegend Precision Counting Beads. Data represents one experimental replicate, 4-5 mice/group.

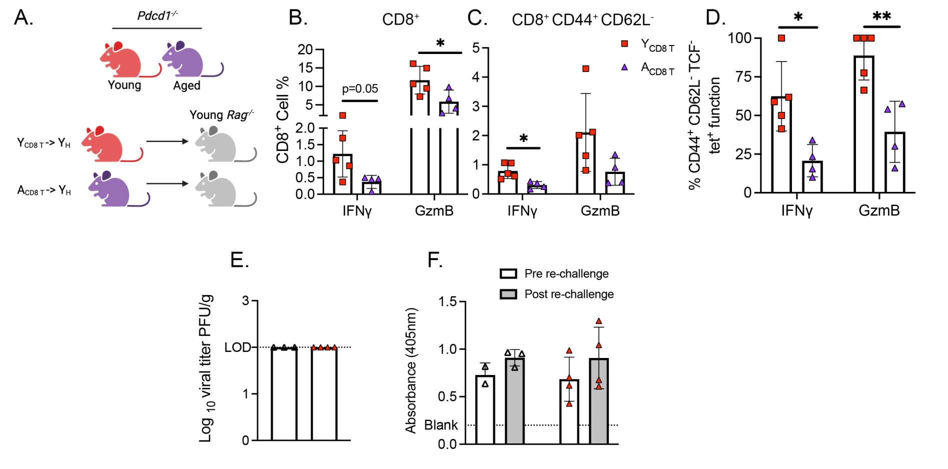

G.

Cell Marker	Fluorophore	Species	Catalog Number	Clone
Both plates				
CD19	BV785	rat	115543	6D5
CD3e	BUV395	hamster	565992	145-2C11
CD4	AF700	rat	100536	RM4-5
CD44	APC-Cy7	rat	560568	IM7
CD62L	BUV563	rat	741230	MEL-14
CD8a	AF532	rat	58-0081-80	53-6.7
Tetramer plate only				
Foxp3	PerCP-Cy5.5	rat	45-5773-82	FJK-16s
T-bet	PE	mouse	644809	4B10
GATA-3	BV711	mouse	565449	L50-823
Rorγt	AF647	mouse	562682	Q31-378
EOMES	AF488	mouse	53-4875-82	Dan11mag
TOX	eFluor™660	mouse	50-6502-82	TXRX10
TCF-7/TCF-1	R718	mouse	567587	S33-966
BCL6	BUV661	mouse	568062	K112-91
PD-1 (CD279)	PE-Cy7	rat	109110	RMP1-30
TIM-3 (CD366)	BV605	rat	119721	RMT3-23
LAG-3 (CD223)	BUV805	rat	748540	C9B7W
2B4 (CD244.1)	BUV737	rat	749155	C9.1
HMPV M94 Class I tetramer	BV421	--	From NIH	--
HMPV N11 Class I tetramer	APC	--	From NIH	--
Peptide stimulation plate only				
Perforin	FITC	rat	11-9392-82	eBioOMAK-D
Granzyme B	PE Cy5.5	Rat	35-8898-80	NGZB
TNFα	BUV661	rat	750025	MIH44
IFNγ	BV650	rat	505831	XMG1.2
CD107a (LAMP1)	PE	rat	121611	1D4B
IL-2	APC	rat	503810	JES6-5H4

Supplemental Figure 4. Young *Pdcd1*$^{-/-}$ CD8$^+$ T cells produce more granzyme B in CD8$^+$ adoptive transfer model.
(A) Experimental schematic for *Pdcd1*$^{-/-}$ CD8$^+$ T cells adoptive transfer. **(B)** IFN and granzyme B expression on bulk CD8$^+$ T cells and **(C)** CD44$^+$ CD62L$^-$ CD8$^+$ T cells. **(D)** Percent functional CD44$^+$ CD62L$^-$ TCF$^-$ tet$^+$ CD8$^+$ T cells. Calculated by percent of CD44$^+$ CD62L$^-$ CD8$^+$ IFN$^+$ or Gzmb$^+$ divided by TCF$^-$ tet$^+$ percent. **(E)** Viral titer in PFU/g in aged isotype and PD-1 blockade groups at day 7 post re-challenge. **(F)** HMPV ELISA absorbance from serum collected by submandibular bleed pre and post re-challenge. **(G)** Table of flow cytometry antibodies. *P<0.05; **P<0.01, ***P<0.001; P<0.0001, unpaired t-test or one-way ANOVA.Data in (A-D) represents one experimental replicate, 4-5 mice/group. Data in (E-F) represents one experimental replicate 3-4 mice/group.
